# Fruit function beyond dispersal: effect of fruit decomposition on the plant microbiome assembly

**DOI:** 10.1111/nph.70698

**Published:** 2025-11-17

**Authors:** Daniel Hoefle, Dinesh Kumar Ramakrishnan, Marie‐Antoinette Holländer, Denis Kiplimo, William Konzag, Leonardo Schena, Antonino Malacrinò, Ayco J. M. Tack, Ahmed Abdelfattah

**Affiliations:** ^1^ Leibniz Institute for Agricultural Engineering and Bioeconomy Max‐Eyth Allee 100 14469 Potsdam Germany; ^2^ Institute for Biochemistry and Biology University of Potsdam Karl‐Liebknecht‐Str. 24/25 14476 Potsdam Germany; ^3^ Department of Agriculture Università degli Studi Mediterranea di Reggio Calabria 89124 Reggio Calabria Italy; ^4^ Department of Biological Sciences Clemson University Clemson SC 29634 USA; ^5^ Department of Ecology, Environment and Plant Sciences Stockholm University 10691 Stockholm Sweden

**Keywords:** carposphere, fruit decomposition, microbial inheritance, phyllosphere, plant microbiome assembly, rhizosphere, seed microbiome phenotype

## Abstract

- The evolutionary role of fruits has primarily been linked to seed dispersal. However, their influence on the soil and plant microbiomes subsequent to their decomposition has received no attention. We hypothesized that fruit decomposition alters the soil microbiome, and consequently the plant microbiome and performance.
- We used amplicon sequencing to analyze the bacterial communities in the soil, rhizosphere, and phyllosphere of tomato and chili plants grown with and without their fruit.
- Fruit decomposition affected soil chemistry, increased bacterial diversity and influenced bacterial community composition. *Blrii41* and *Sandaracinaceae* and functions related to methanol oxidation and nitrification, mammalian and human gut metabolism were enriched. It also decreased germination rates and affected shoot but not root length. Fruit decomposition decreased phyllosphere microbial diversity and strongly shifted the rhizosphere and phyllosphere community composition. The plant microbiome showed increased functions related to ligninolysis, methanol oxidation, methylotrophy, and xylanolysis, among others.
- These results provide evidence that fruits exert a postdispersal influence on the seedling environment and the early plant microbiome assembly. This study expands the classical ecological view of fruit function and opens new directions for understanding microbial inheritance and leveraging fruit‐derived microbiomes.

## Introduction

**Fig. 1:**
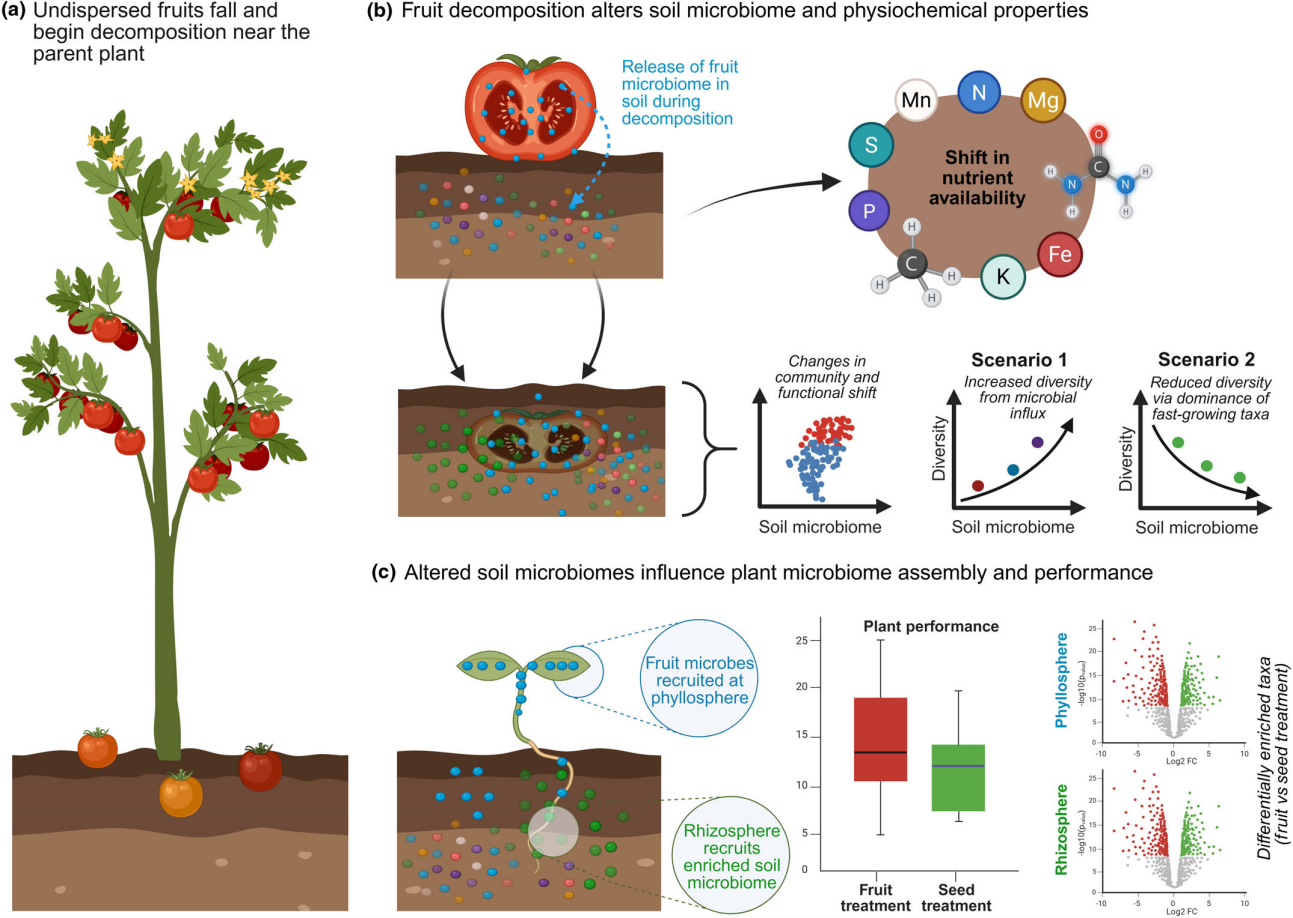
Impact of fruit decomposition on soil and plant microbiomes. (a) Fruits harbor their own microbiome, which is released into the soil upon decomposition. (b) Fruit decomposition changes the soil microbiome by introducing fruit‐associated microbes and altering microbial interactions. (c) As seeds germinate, the enriched soil microbiome colonizes the emerging plant, influencing the microbial assembly in roots and shoots. This interplay between fruit decomposition, soil microbial dynamics, and plant colonization suggests that fruits may play an active role in shaping the microbiome of the next plant generation beyond seed dispersal. This figure was created in BioRender (https://BioRender.com/tw7zbn6).

Angiosperms have evolved fleshy fruits primarily to facilitate seed dispersal by frugivores, also known as zoochory (Eriksson, 2016). Since their evolution *c*. 100 million years ago, plants developed a wide range of fruit traits, including, but not limited to, shape, color, and aromatic volatiles to attract frugivorous animals (Fenner, 2000; Albrecht *et al*., 2012). Fruit production requires a substantial amount of energy and can account for a large fraction of plant biomass (Pickering & Arthur, 2003; Seymour *et al*., 2013). However, not all fruits are successfully dispersed, and many fall down and decompose on the ground near the parent plant (Fig. 1a). While the evolutionary role of fruits in seed dispersal is well established, their alternative ecological and evolutionary functions remain largely unexplored (Albrecht *et al*., 2013; Eriksson, 2016).

Similar to other plant organs, fruits are associated with a diverse collection of microorganisms shaped by several biotic and abiotic factors (Wassermann *et al*., 2019; Kusstatscher *et al*., 2020; Piombo *et al*., 2020; Abdelfattah *et al*., 2021; Olimi *et al*., 2022; Zhimo *et al*., 2022; Bartuv *et al*., 2023). Although the fruit microbiome has been less studied compared to the phyllosphere and rhizosphere, little is known about the microbial community dynamics during decomposition, or how these changes influence soil and emerging seedling microbiomes (Fig. 1b). These questions are crucial for our understanding of the sources and assembly mechanisms of the plant microbiome (Compant *et al*., 2010; Truyens *et al*., 2015; Shade *et al*., 2017; Berg & Raaijmakers, 2018; Abdelfattah *et al*., 2022; Hoefle *et al*., 2024). Plants inherit a small fraction of their microbiome through seeds and are subsequently colonized by environmental microorganisms, mainly from the soil (Wassermann *et al*., 2022). This means that variations in the species pool at the germination stage can significantly influence the seedling microbiome (Fig. 1c) (Rochefort *et al*., 2021). If fruit decomposition plays a role in structuring the plant microbiome and its functions, this could represent an overlooked ecological and evolutionary role of fruits: fruits could influence plant functioning as well as provide a mechanism for microbial inheritance.

We tested two contrasting fruit types to evaluate the effects of fruit decomposition: *Solanum lycopersicum* (tomato) and *Capsicum annuum* (chili). Tomatoes are a typical fleshy fruit rich in sugars, organic acids, and antioxidant compounds that may benefit a large number of microorganisms during decomposition (Ali *et al*., 2021). By contrast, chili fruits contain capsaicinoids, which have antimicrobial properties that could suppress certain microbial taxa (Hamed *et al*., 2019), potentially leading to distinct microbiome assembly patterns in the soil. This contrast provides an opportunity to explore whether fruit chemistry influences the soil microbiome differently as well as the generality of the role of fruit decomposition in the assembly of the plant microbiome and microbial inheritance. Specifically, we addressed three key research questions:

1. What is the effect of fruit decomposition on soil chemistry and microbiome?
2. How does fruit decomposition affect seed germination and plant growth?
3. What is the effect of fruit decomposition on the diversity, composition, and functioning of the soil, root, and leaf microbiomes?

We expect that fruit decomposition changes the composition of the soil microbiome due to the introduction of additional resources, such as water and organic matter. In terms of microbial diversity, we foresee one of two scenarios: (1) fruit decomposition may result in increased microbial diversity due to the introduction of fruit‐associated microbes to the soil or (2) a reduction in microbial diversity due to the enrichment of certain microbial taxa, particularly fast‐growing bacteria (Fig. 1b).

Changes in the soil microbiome are likely to influence the assembly of the emerging seedling phyllosphere and rhizosphere microbiome (Fig. 1c) due to alterations in the pool of species from which the plant recruits its microbiome (Shade *et al*., 2017; Trivedi *et al*., 2020; Wolfgang *et al*., 2023). Moreover, these changes in the microbiome are likely to be accompanied by shifts in microbial functions. Changes in the soil or plant microbiome may affect seed germination and seedling phenotypes, potentially benefiting seedling growth by increasing the abundance of plant‐beneficial microbes or, conversely, detrimental through the proliferation of pathogenic or antagonistic microorganisms (Berg & Smalla, 2009; Mendes *et al*., 2013; Berg *et al*., 2021). Given that fruits also release nutrients into the soil, we anticipate that changes in plant microbiomes may be accompanied by differences in seedling growth and phenotypes. However, the direction of these effects may depend on fruit chemistry while decomposing tomato fruits may primarily act as a microbial and nutrient source, chili fruits, due to their capsaicin content, might suppress certain microbial groups, potentially leading to distinct microbiome assembly patterns and plant performance outcomes.

## Materials and Methods

To assess the effect of fruit decomposition, we planted cherry tomatoes (*Solanum lycopersicum* L.) and chili (*Capsicum annuum* L.) seeds with and without their fruits, hereafter referred to as *fruit treatment* and *seed treatment*. Both experiments were conducted independently, with the chili experiment serving to confirm patterns observed in the tomato experiment, while including additional analysis of physicochemical soil properties. We acknowledge the technical differences between the two experiments, yet we want to emphasize the consistent patterns observed across two taxonomically and physiologically distinct plant species. To minimize genetic variation among seeds, which in turn may influence the plant microbiome and phenotype, seeds used in both treatments were collected from the same fruit. Fruits were cut in half, and one half was placed in soil at a depth of 1 cm including 5 seeds. In our experiments, we buried the fruits and seeds to isolate the effect of fruit decomposition while minimizing potential bias from differences in germination timing, since placing seeds and fruits on the soil surface might alter germination kinetics. Given the rapid turnover of soil microbial communities, seedlings in different treatments could have been exposed to different soil microbiomes. Five seeds from the other half were separated from the pulp, rinsed with sterile deionized water, dried at 30°C for 24 h, and planted in soil at a depth of 1 cm. In the tomato experiment we used 4 × 4 × 5.5 cm (L × W × H) pots, with a total of 10 pots and 60 plants for the fruit treatment and 10 pots and 50 plants in the seed treatment. In the chili experiment we used 5 × 5 × 6 cm (L × W × H) pots, with a total of 162 pots and 810 plants. For soil microbiome analysis at each time point 10 soil replicates per treatment were sampled within a 0.5 cm radius around the sowing site or emerging plant, ensuring proximity to the root zone and capturing the relevant microbial environment. To analyze the seedling microbiome at harvest 10 random plants of each treatment were selected. We used organic soil (Soil for tomatoes and vegetables, Floragard, Oldenburg, Germany) for all plants. All plants were cultivated at 25°C in a controlled environment equipped with artificial light (FLUXshield 300; Crescience, Munich, Germany) under 16 h light : 8 h dark.

### Sampling and processing of samples

To monitor the dynamics of the soil microbiome community during fruit decomposition, we collected soil samples at three time points. Sowing (T0), 6 d after sowing and before seed germination (T1), and 18 d after sowing (T2). At T2, seedlings were harvested and separated into above and belowground parts, hereafter referred to as *phyllosphere* and *rhizosphere*. To measure the length of shoots and roots, the seedlings were carefully extracted from the soil and gently shaken to eliminate excess soil adhering to the roots. Subsequently, the collected roots were used for rhizosphere microbiome analysis and to measure root length. The roots were detached from the shoot using sterilized scissors and rinsed in sterile dH_2_O to eliminate the loosely adhered soil while retaining the firmly attached soil for rhizosphere analysis. The collected shoots were subjected to surface sterilization by immersion in 0.5% sodium hypochlorite for 1 min and washed three times with sterile dH_2_O. In this study, we define the phyllosphere as the shoot endosphere microbial community, and the rhizosphere as a mixture of rhizosphere, rhizoplane, and endophytic microorganisms.

### Soil physicochemical analysis

Analysis of soil physicochemical properties was only performed in the chili soil. Therefore, we added an additional 12 replicates for each treatment. We analyzed three time points under fruit and seed treatments to determine whether fruit decomposition influences nutrient availability. At time points T0, T1, and T2, four replicates of *c*. 150 g of soil were collected from the bulk soil for analysis for each treatment. The sown chili fruits and seeds were kept inside the soil during sampling, and unless otherwise stated, they remained in the soil during nutrient measurements or extraction. After germination, fruits and plants were removed from the soil. The soil samples were stored at −20°C until analysis. The soil analysis was conducted by the Central Analytics Department at the Leibniz Institute for Agricultural Engineering and Bioeconomy in Potsdam, Germany. Detailed information regarding the soil analysis is available in the Supporting Information (Notes S1).

### DNA extraction, amplicon library preparation, and sequencing

To investigate the bacterial community and diversity of soil and plant samples, DNA was extracted from all samples using the MP DNA Spin Kit for Soil (MP Biomedicals, CA, USA), according to the manufacturer's instructions. Extracted DNA was used to amplify the 16S V4 gene region using universal primers 515f and 806r and peptide nucleic acid clamps (PNA) to interfere with host plastid and mitochondrial DNA amplification. Subsequently, the DNA fragments were purified using magnetic bead chemistry and sequenced on an Illumina platform. Detailed information on sample preparation, amplicon library generation, sequencing, and sequence pre‐processing is available in Notes S2.

### Statistical analysis

#### Statistical analysis of soil physicochemical properties

To assess the impact of fruit decomposition on soil abiotic properties, we analyzed a range of chemical parameters across three time points (T0, T1, and T2) for both fruit and seed treatments. The differences between treatments across time points were evaluated using *emmeans_test* from the rstatix package (Alboukadel, 2023), with a Bonferroni correction applied for multiple testing.

#### Germination rate and seedling growth assessment

Germination success was measured at the time of harvest (18 d after sowing) as the proportion of seeds germinated per pot. Each pot contained multiple seeds, five seeds in the seed treatment and six in the fruit treatment for tomatoes and five seeds for both treatments in chili. To evaluate the effect of fruit decomposition on seedling growth, we measured shoot length, and root length. To analyze treatment effects on germination rate, we fitted a generalized linear model (GLM) with a binomial distribution and logit link function (McCullagh & Nelder, 2019), using the number of germinated and nongerminated seeds per pot as the response variable, and pairwise comparisons of estimated marginal means were performed using the emmeans package (Russell, 2025). For shoot and root length, pairwise statistical comparisons between treatments were conducted using the *emmeans_test* function from the rstatix package (Alboukadel, 2023), with Bonferroni correction applied for multiple testing.

#### Microbial community analysis

To test the effect of fruit decomposition and time on soil microbial diversity, we modeled species richness and Shannon diversity as a function of the fixed effects *treatment*, *time*, and *their interactions* using ANOVA from the car package (Fox & Weisberg, 2020). To test the effect of fruit decomposition and time, we modeled the multivariate microbial community composition as a function of *the interaction of treatment* and *time* using a PERMANOVA with the *adonis2* function in the vegan package (Oksanen *et al*., 2013). For the phyllosphere and rhizosphere microbiome a separate PERMANOVA was applied on each plant compartment to test the effect of fruit decomposition on the community composition.

#### Taxonomic differential analysis

To identify the enriched bacterial taxa between fruit and seed treatment, we used Linear Discriminant Analysis Effect Size (LEfSe) (Segata *et al*., 2011). Separate LEfSe analyses were performed at the genus level for soil at T2, rhizosphere, and phyllosphere. Taxa with an adjusted *P*‐value <0.05 were considered significantly enriched.

#### Functional differential analysis

Cumulative sum scaling‐normalized data were used to infer the potential functional roles of bacterial communities using Functional Annotation of Prokaryotic Taxa (FAPROTAX) (Louca *et al*., 2016). We used FAPROTAX to obtain a first, hypothesis‐generating view of putative functions. FAPROTAX maps taxa to functions based on literature on cultured representatives and was originally developed for marine datasets. Therefore, coverage and accuracy can be limited for soil‐ and plant‐associated functions. Accordingly, assignments should be interpreted as potential capacities rather than evidence of activity. To test the effect of fruit decomposition on soil, phyllosphere and rhizosphere functional compositions, we used PERMANOVA as described above. To identify microbial functions that significantly differed between treatments, we conducted differential abundance analysis using DESeq2 (Love *et al*., 2014). Functional profiles from fruit and seed treatment microbiomes were compared across all compartments, with significant log_2_ fold change (LFC) thresholds and an adjusted *P*‐value < 0.05. For tomatoes, functions with a |LFC| ≥ 1 in soil at T2, |LFC| ≥ 0.5 in the phyllosphere, and |LFC| ≥ 2.0, in the rhizosphere were considered significantly enriched. In chili, thresholds were set as |LFC| ≥ 0.5 for soil at T2, and |LFC| ≥ 1.0 for both the phyllosphere and rhizosphere. We used different LFC thresholds to reflect the strength and variability of the treatment effects in each plant compartment. Significant pathways were visualized using volcano plots.

#### SourceTracker2 analysis

To assess the potential origins of microbial communities in tomato and chili seedlings, we performed a SourceTracker2 analysis (Knights *et al*., 2011). The phyloseq object was rarefied to 500 sequences per sample using the *rarefy_even_depth* function in the phyloseq package. The ASV table was extracted and converted to BIOM format. The analysis was run in Ubuntu using the Gibbs sampling implementation of SourceTracker2, with sample type defined by the SourceSink column in the metadata and environmental categories provided in the Env column, that is soil, phyllosphere, rhizosphere, fruit, and seed. Soil_T0, Soil_t1, Soil_T2, and seed and fruit, respectively, were defined as a source, and phyllosphere and rhizosphere as a sink.

## Results

### Fruit decomposition affects soil physicochemical properties

**Fig. 2:**
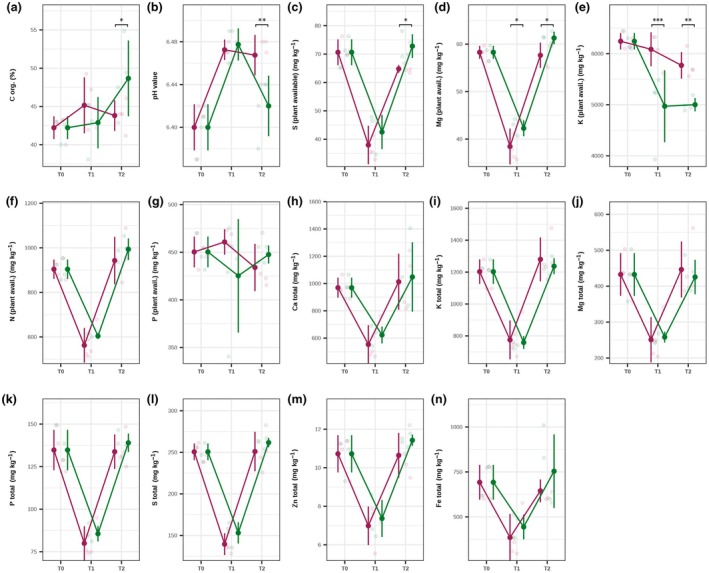
Effect of fruit decomposition on soil physicochemical properties and nutrient availability in chili (*Capsicum annuum* L.) cultivation. (a–n) Changes in soil organic carbon (C org.), pH, plant‐available sulfur (S), magnesium (Mg), potassium (K), nitrogen (N), phosphorus (P), and total elemental concentrations of Ca, K, Mg, P, S, Zn, and Fe across sampling times (T0, T1, and T2) under fruit and seed treatments are shown. Points represent mean values per treatment; vertical lines indicate ± SD. Significant differences between treatments (*P* < 0.05; Bonferroni‐adjusted emmeans pairwise comparisons) are indicated by black asterisks above the corresponding time points. Purple, fruit treatment; green, seed treatment.

In soil under chili cultivation, physicochemical properties and nutrient availability showed temporal changes in both the fruit and seed treatments (Fig. 2; Table S1). Organic carbon increased significantly over time (FC = 1.11, Table S1), with a more pronounced increase in the seed treatment (*P* = 0.04). Soil pH displayed a transient increase at T1, followed by a significant decline at T2 in the seed treatment (*P* = 0.0035, H^+^ FC = 1.12, Table S1). While plant‐available phosphorus was relatively stable over time with no treatment effect, plant‐available sulfur (FC = 1.12, Table S1) and magnesium (FC = 1.06, Table S1) were significantly higher in the seed treatment. Only plant‐available potassium exhibited a significant reduction in the seed treatment (FC = 0.87, Table S1) compared to the fruit treatment. The total elemental content of magnesium, calcium, potassium, sulfur, zinc, iron, and phosphorus showed transient depletion at T1, with partial recovery at T2, without significant treatment effects (Table S1).

### Microbial and functional shifts in the soil through fruit decomposition

**Fig. 3:**
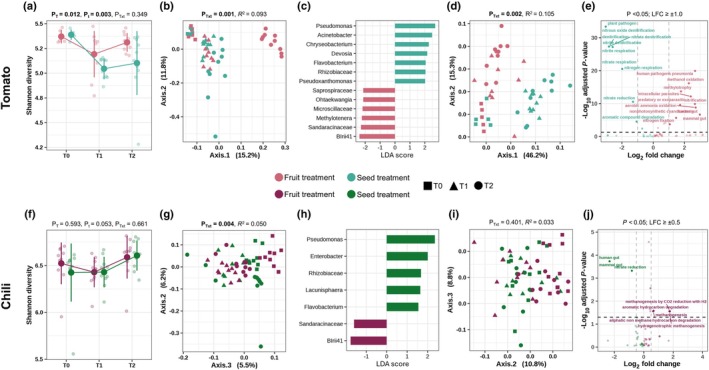
Effect of fruit decomposition on soil‐ and plant‐associated microbial diversity, composition, and function in tomato (*Solanum lycopersicum* L., upper panel) and chili (*Capsicum annuum* L., lower panel). (a, f) Shannon diversity of soil bacterial communities over time (T0–T2) under fruit and seed treatments; error bars represent ±SD; *P*‐values for Shannon diversity were obtained by ANOVA investigating the effect of treatment (P_T_), time (P_t_), and their interaction (P_Txt_). (b, g) Principal coordinate analysis (PCoA, Bray–Curtis distances) of community composition; P‐ and *R* ^2^ values derived from PERMANOVA testing the interactive effect of treatment and time (P_Txt_). (c, h) Linear discriminant analysis (LDA) Effect Size (LEfSe) plots showing differentially enriched bacterial taxa resolved to the genus level under fruit and seed treatments in soil with an LDA score (log_2_ fold change) > 2.0); in cases where genus‐level classification was unavailable, the corresponding family‐level taxa are shown. (d, i) PCoA of predicted functional composition based on FAPROTAX profiles at different time points (T0–T2); significance assessed by PERMANOVA testing the interactive effect of treatment and time (P_Txt_). (e, j) Volcano plots of predicted bacterial functions showing significantly enriched functions (adjusted *P* < 0.05) with log_2_ fold change (LFC) thresholds of ≥ 1.0 for tomato and ≥ 0.5 for chili indicated by dashed lines. Partial *R* ^2^ values indicate the proportion of variation explained by treatment or time. Purple, fruit treatment; green, seed treatment; symbols (■, ▲, ⚫), denote sampling times T0, T1, T2.

In tomatoes, fruit decomposition and time had a significant effect on soil microbial diversity (*P* = 0.012 and *P* = 0.003), but not their interaction (*P* = 0.349) (Fig. 3a; Table S2). However, fruit decomposition, time, and their interaction had no significant effects on soil microbial diversity in chili (*P* = 0.593; *P* = 0.053; *P* = 0.661) (Fig. 3f; Table S2). The interaction of fruit decomposition and time of both tomato and chili fruits had a significant effect on the soil microbial community composition (*P* = 0.001 and *P* = 0.004) (Fig. 3b,g; Table S2). Interestingly, at T2, both tomato and chili fruit treatments showed enrichment of the same genera, *Blrii41* and *Sandaracinaceae*. In tomato, the seed treatment was enriched in *Pseudomonas*, *Acinetobacter*, *Chryseobacterium*, *Devosia*, *Flavobacterium Rhizobiaceae* family, and *Pseudoxanthomonas*, whereas in the chili seed treatment, *Pseudomonas, Enterobacter, Rhizobiaceae* family, *Lacunisphaera*, and *Flavobacterium* were enriched (Fig. 3c,h).

Functional prediction of the soil microbiome mirrored changes in the community composition. In tomatoes, the microbial functional potential significantly differed between treatments (*P* = 0.002) (Fig. 3d; Table S3). The fruit treatment had a significantly higher relative abundance of functions associated with mammalian and human gut metabolism, methanol oxidation, and nitrification in the soil at T2, whereas the seed treatment soil at T2 was enriched in nitrogen cycling pathways, including nitrate respiration, denitrification, nitrite reduction, and nitrous oxide denitrification (Fig. 3e; Table S3). In chili soil, the functional composition did not change significantly by treatment (*P* = 0.401) (Fig. 3i; Table S3). However, in chili, the fruit treatment significantly enriched methanogenesis, methanogenesis by CO_2_ reduction with H_2_, aliphatic nonmethane hydrocarbon degradation, hydrogenotrophic methanogenesis, and aromatic hydrocarbon degradation, whereas in the seed treatment, the soil at T2 was enriched in gut‐associated functions (Fig. 3j; Table S3).

### Fruit decomposition decreases germination rate

**Fig. 4:**
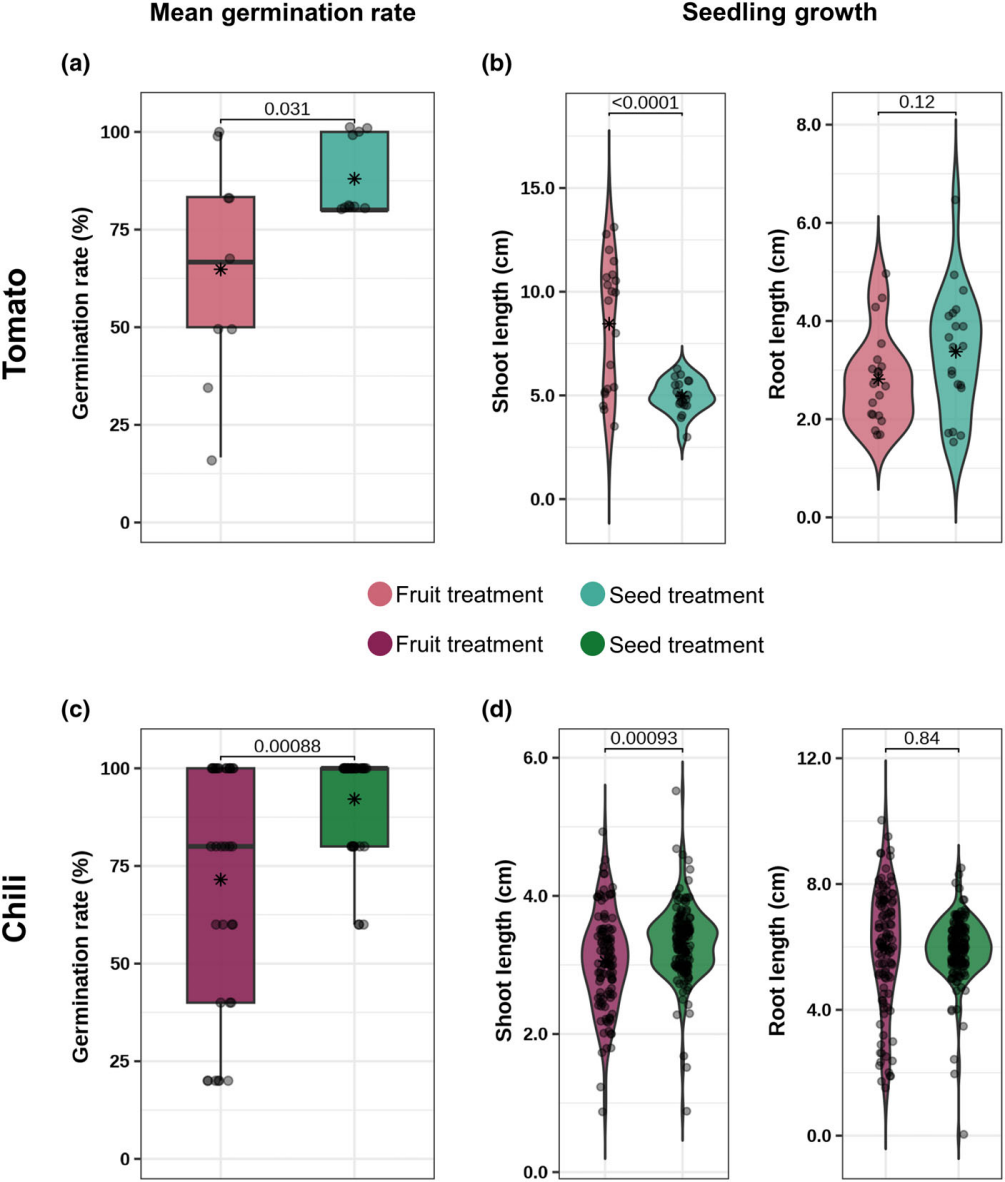
Effect of fruit decomposition on germination and early growth of tomato (*Solanum lycopersicum* L., upper panel) and chili (*Capsicum annuum* L., lower panel) seedlings. (a, c) Mean germination rate (%) under fruit and seed treatments for tomato (upper panel) and chili (lower panel). Boxplots show medians (horizontal line), interquartile ranges (boxes), and whiskers extending to 1.5 × IQR; the mean is indicated by a black asterisk. *P*‐values are from pairwise comparisons with Bonferroni adjustment. (b, d) Seedling growth performance represented by shoot and root length (cm) under the two treatments. Violin plots display kernel‐density envelopes with individual observations (jittered dots) and the mean marked by a black asterisk. Purple, fruit treatment; green, seed treatment.

The germination rate was significantly lower in the fruit treatment compared to the seed treatment for both tomato (*P* = 0.031) and chili (*P* = 0.00088) plants (Fig. 4a,c). In tomatoes, shoot length was significantly higher in the fruit treatment (P ≤ 0.0001), whereas root length did not differ between treatments (*P* = 0.12) (Fig. 4b). In chili, the fruit treatment shoot length was significantly lower than that in the seed treatment (*P* = 0.00093), and root length showed no significant differences between treatments (*P* = 0.84) (Fig. 4d).

### Changes in microbial diversity and composition in phyllosphere and rhizosphere microbiomes

**Fig. 5:**
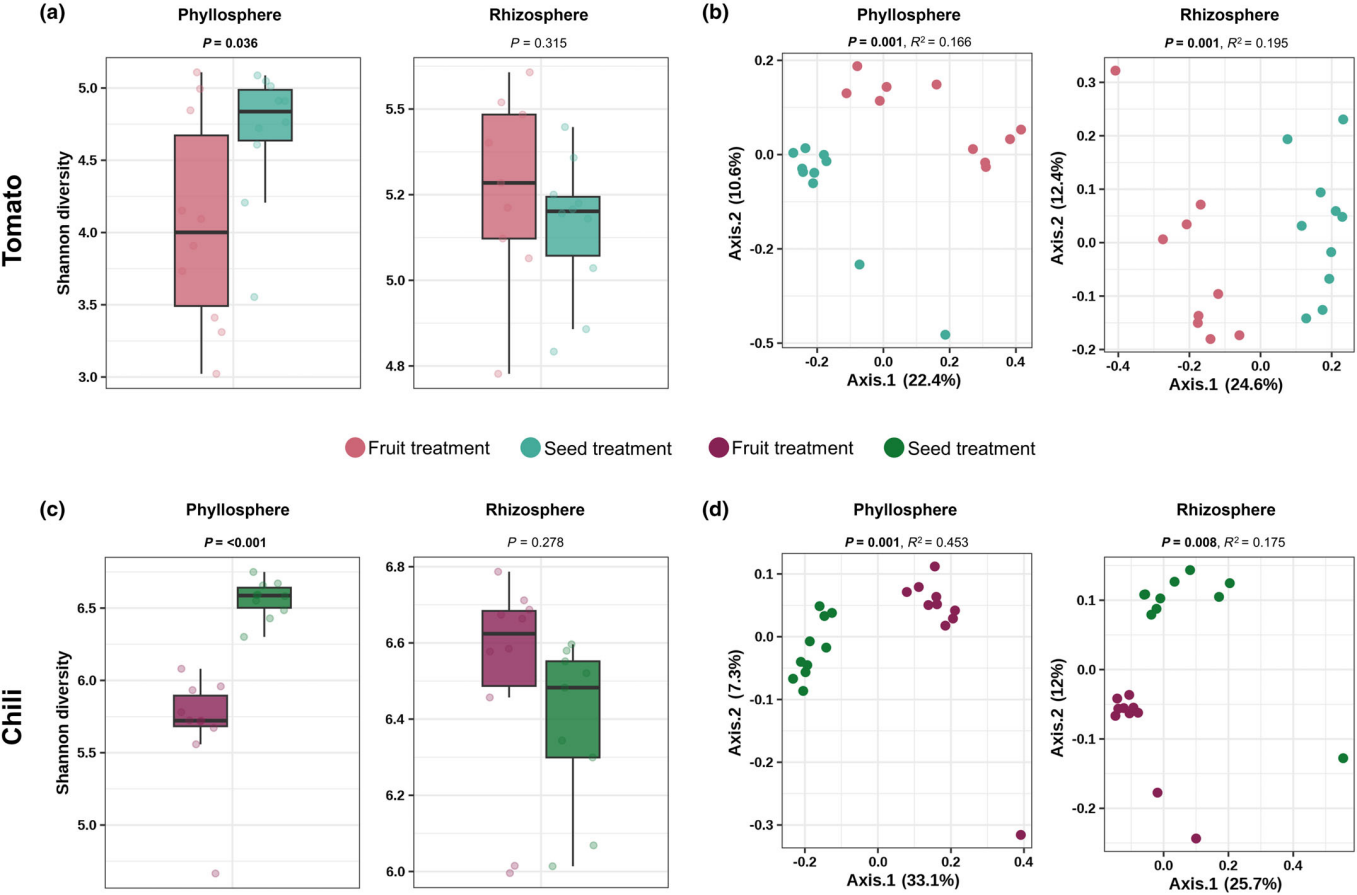
Effect of fruit decomposition on phyllosphere and rhizosphere microbial diversity and community composition in tomato (*Solanum lycopersicum* L., upper panel) and chili (*Capsicum annuum* L., lower panel). (a, c) Shannon diversity of bacterial communities in the phyllosphere and rhizosphere under fruit and seed treatments. Boxplots display medians (horizontal lines), interquartile ranges (boxes), and whiskers extending to 1.5 × IQR; dots represent individual samples. *P*‐values were obtained from ANOVA (b, d) Principal coordinate analysis (PCoA; Bray–Curtis distances) showing community composition by treatment. *P*‐values were calculated using PERMANOVA. Purple, fruit treatment; green, seed treatment.

In both tomato and chili, the seedling phyllosphere microbiome from the fruit treatment had a significantly lower microbial diversity (*P* = 0.036 and *P* < 0.001) compared to the phyllosphere of the seed treatment (Fig. 5a,c; Table S4). However, the rhizosphere microbial diversity was not significantly affected by the treatment (*P* = 0.315 and *P* = 0.278) (Fig. 5a,c; Table S4). The microbial community composition in both the phyllosphere and rhizosphere of tomato and chili differed significantly between seed and fruit treatments (tomato: phyllosphere: *P* = 0.001, *R* ^2^ = 0.166; rhizosphere: *P* = 0.001, *R* ^2^ = 0.195; chili: phyllosphere: *P* = 0.001, *R* ^2^ = 0.453; rhizosphere: *P* = 0.008, *R* ^2^ = 0.175) (Fig. 5b,d; Table S4).

### Key microbes driving changes in the seedling microbiome

**Fig. 6:**
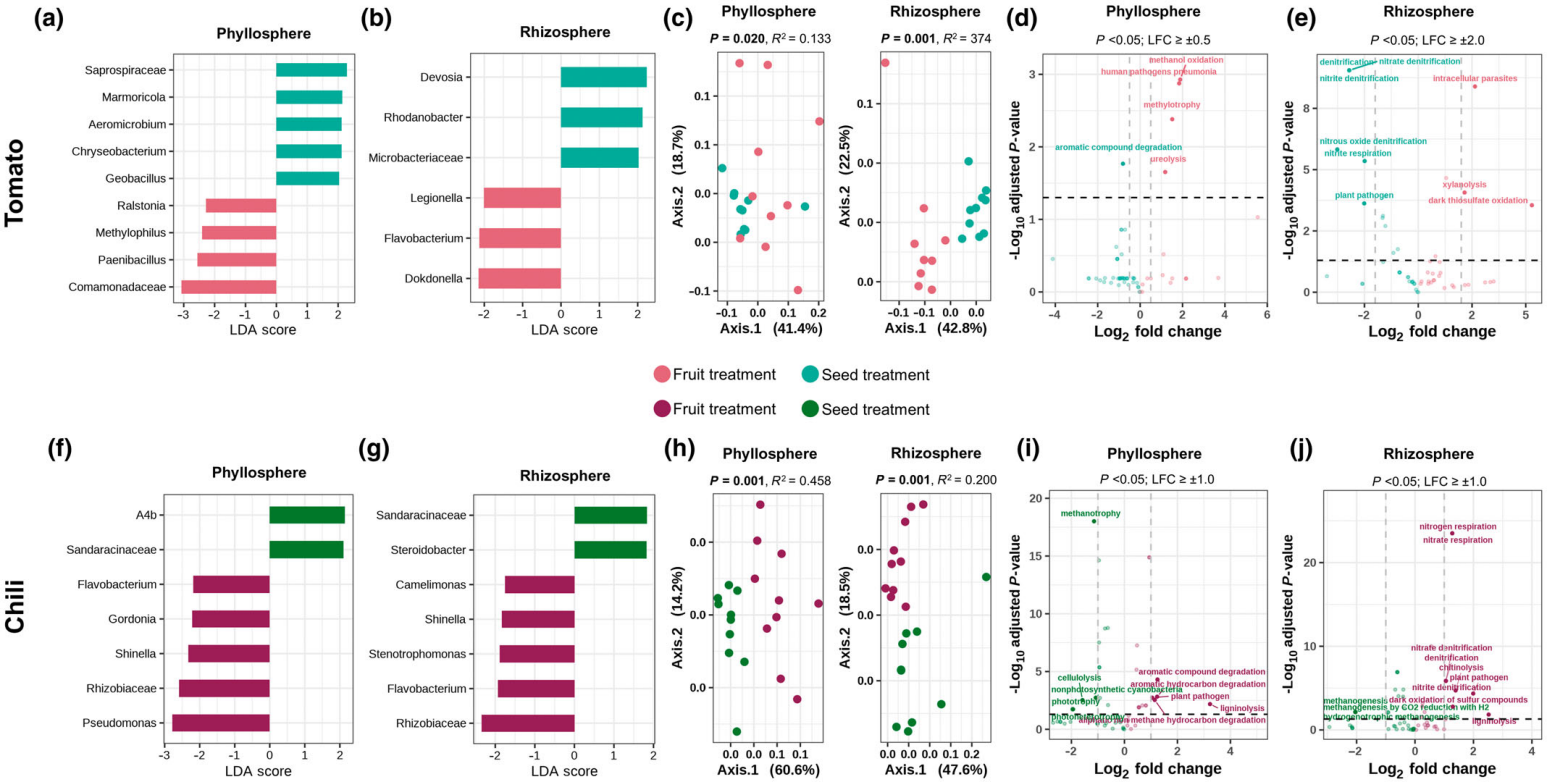
Effect of fruit decomposition on phyllosphere and rhizosphere microbial composition and functional potential in tomato (*Solanum lycopersicum* L., upper panel) and chili (*Capsicum annuum* L., lower panel). (a, b, f, g) Linear discriminant analysis effect size (LEfSe) plots display differentially abundant bacterial taxa resolved to the genus level under fruit and seed treatments in the phyllosphere and rhizosphere with an Linear Discriminant Analysis (LDA) score (log_2_ fold change) > 2.0; in cases where genus‐level classification was unavailable, the corresponding family‐level taxa are shown. (c, h) Principal coordinate analysis (PCoA; Bray–Curtis distances) depicting functional community composition across treatments and sampling times (T0–T2); *P*‐values and partial *R* ^2^ values were derived from PERMANOVA. (d, e, i, j) Volcano plots of predicted bacterial functions showing significantly enriched functions (adjusted *P* < 0.05) with Log_2_ fold‐change (LFC) thresholds indicated by dashed lines. Purple, fruit treatment; green, seed treatment.

The phyllosphere of the tomato fruit treatment was enriched in *Comamonadaceae*, *Paenibacillus*, *Methylophilus*, and *Ralstonia*, whereas the seed treatment had a higher abundance of *Saprospiraceae, Marmoricola, Aeromicrobium*, *Chryseobacterium*, and *Geobacillus* (Fig. 6a). In the rhizosphere, the tomato fruit treatment showed enrichment of *Dokdonella*, *Flavobacterium*, and *Legionella*, whereas the seed treatment showed a higher abundance of *Devosia*, *Rhodanobacter*, and *Microbacteriaceae* (Fig. 6b). In chili, the phyllosphere of the fruit treatment was enriched in *Pseudomonas*, *Rhizobiaceae* family, *Shinella*, *Gordonia*, and *Flavobacterium*, while the seed treatment was enriched in *A4b* and *Sandaracinaceae* family (Fig. 6f). A similar pattern was observed in the rhizosphere, where *Sandaracinaceae* and *Steroidobacter* were enriched in the seed treatment, whereas the fruit treatment showed a higher abundance of *Rhizobiaceae* family, *Flavobacterium*, *Stenotrophomonas*, *Shinella*, and *Camelimonas* (Fig. 6g).

Similar to the community composition of the phyllosphere and rhizosphere microbiomes, the functional composition also reflected significant changes between fruit and seed treatments in both tomato and chili phyllosphere and rhizosphere (tomato: phyllosphere: *P* = 0.020, *R* ^2^ = 0.133; rhizosphere: *P* = 0.001, *R* ^2^ = 0.374; chili: phyllosphere: *P* = 0.001, *R* ^2^ = 0.458; rhizosphere: *P* = 0.001, *R* ^2^ = 0.200) (Fig. 6c,h; Table S5). In tomato, the phyllosphere of the fruit treatment displayed significant enrichment in functions related to methanol oxidation, methylotrophy, ureolysis, and human pathogen metabolism, whereas the seed treatment had a higher abundance of aromatic compound degradation functions (Fig. 6d; Table S3). In the tomato rhizosphere, fruit treatment enriched xylanolysis and dark thiosulfate oxidation, while the seed treatment had a higher abundance of nitrogen cycling functions, including denitrification, nitrite respiration, and nitrous oxide denitrification (Fig. 6e; Table S3). The phyllosphere of the fruit treatment in chili was enriched in aromatic hydrocarbon degradation, plant pathogen metabolism, and ligninolysis, whereas the seed treatment showed a higher representation of methanotrophy (Fig. 6i; Table S3). In the chili rhizosphere, fruit treatment was significantly associated with higher abundances of nitrate respiration, denitrification, chitinolysis, and sulfur oxidation, while the seed treatment was enriched in methanogenesis and CO_2_ reduction (Fig. 6j; Table S3).

### Fruit as a source of seedling microorganisms

**Fig. 7:**
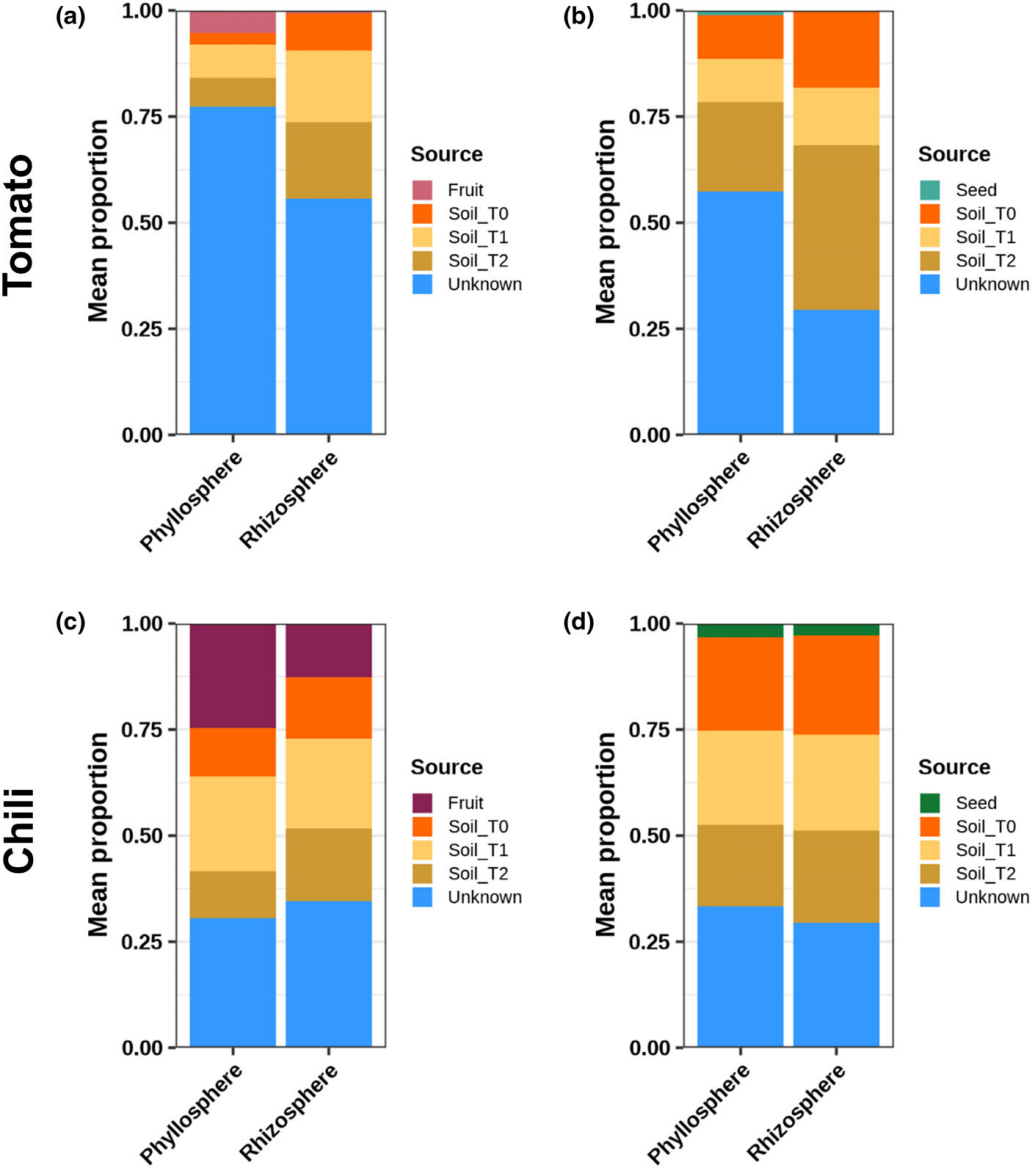
SourceTracker2 analysis of microbial source contributions to the phyllosphere and rhizosphere of tomato (*Solanum lycopersicum* L., upper panel) and chili (*Capsicum annuum* L., lower panel) seedlings. Bar plots show the mean proportional contribution of fruit, seed, and soil sources to the microbiomes of the phyllosphere and rhizosphere across sampling times (T0, T1, T2). (a) Tomato fruit treatment, (b) tomato seed treatment, (c) chili fruit treatment, (d) chili seed treatment. Source proportions were inferred using SourceTracker2 (Gibbs sampling) based on 16S rRNA gene amplicon data. Colors indicate source categories: fruit (purple), seed (green), soils at different time points (T0–T2; orange to brown gradient), and unknown sources (blue). Bars represent mean relative contributions across biological replicates. In tomato, soil dominated as the main source, whereas fruit accounted for only a small fraction of the seedling microbiome (phyllosphere = 5.2%, rhizosphere = 0.6%). In chili, fruit contributed substantially more (phyllosphere = 24.6%, rhizosphere = 12.7%), whereas seed treatments showed much smaller inputs (tomato: phyllosphere = 1.2%, rhizosphere = 0.18%; chili: phyllosphere = 3.2%, rhizosphere = 2.4%).

We investigated microbial coalescence from fruit through decomposition into the seedling by quantifying the relative contributions of fruit, seed, and soil as sources of plant‐associated microorganisms over time. In tomato, 5.2% of the microorganisms in the phyllosphere result from the fruit, whereas only 0.6% of the rhizosphere microbiome is explained by the fruit (Fig. 7a; Table S6). In the tomato seed treatment, 1.2% of the microorganisms in the phyllosphere come from the seed, and only 0.18% in the rhizosphere (Fig. 7b; Table S6). By contrast, in chili, 24.6% of the phyllosphere and 12.7% in the rhizosphere microbiome originated from the chili fruit (Fig. 7c; Table S6). The chili seed treatment showed that 3.2% of the phyllosphere and 2.4% of the rhizosphere microbiome originated from the seed (Fig. 7d; Table S6).

## Discussion

### The effect of fruit decomposition on the soil physicochemical properties, the soil microbiome, and predicted functions

Since the tomato and chili experiments were conducted sequentially, with soil physicochemical measurements introduced only in the chili experiment, the following results are limited to chili soils. The only changes we observed in soil with decomposing fruit were in organic carbon, pH, and plant‐available sulfur, magnesium, and potassium. All physicochemical properties except organic carbon, pH, plant‐available potassium, and phosphorus showed transient depletion at T1, with partial recovery at T2. The decrease in elemental contents at T1 is likely related to leaching caused by our watering regime, as samples were collected shortly after watering. The increase in pH and potassium is consistent with the high potassium content of chili fruits (Zhou *et al*., 2024). The decline in organic carbon aligns with a positive priming effect, where labile carbon inputs accelerate mineralization of native soil organic matter (Liu *et al*., 2020). Elevated potassium competes with and displaces magnesium – higher pH promotes magnesium leaching, thereby reducing plant‐available magnesium (Gransee & Führs, 2013). The drop in plant‐available sulfur likely reflects microbial immobilization of sulfate into biomass, a common short‐term response during residue decomposition (Wu *et al*., 1993). However, since only a minority of measured soil properties changed due to fruit decomposition, we suggest that observed shifts in plant fitness are more likely driven by alterations in the plant microbiome rather than changes in soil chemistry.

Functional changes in soil between fruit and seed treatments varied between tomatoes and chili. Fruit decomposition influenced soil microbial diversity differently in the tomato and chili treatments. In tomatoes, both decomposition and time significantly altered microbial diversity, but not their interactions. Neither had an effect on the soil microbial diversity in chili. Tomato soils showed stronger shifts after fruit decomposition than chili soils, consistent with crop‐specific differences in the soil microbiome responses to residue inputs (Prescott & Grayston, 2013; Almagro *et al*., 2021). Moreover, tomato fruits are rich in sugars, flavonoids, and metabolites, which can stimulate microbial activity and promote diversity over time (Escobar Rodriguez *et al*., 2021). By contrast, chili fruits contain antimicrobial capsaicinoids, which may suppress microbial growth (Romero‐Luna *et al*., 2023). Fruit decomposition significantly altered the microbial community composition in both tomatoes and chili. Both fruit treatments displayed enrichment of two taxa, *Blrii41* and *Sandaracinaceae*. Tomato seed treatment enriched *Pseudomonas, Acinetobacter*, and *Chryseobacterium*, whereas in chili seed treatment, *Pseudomonas*, *Enterobacter*, *Lacunisphaera*, and *Flavobacterium* dominated. The shared enrichment of *Blrii41* and *Sandaracinaceae* in both fruit treatments suggests that fruit decomposition creates a selective environment favoring these taxa, and that both are probably involved in degrading fruit‐derived complex carbohydrates (Cai *et al*., 2018; Dai *et al*., 2021). *Pseudomonas* and *Flavobacterium* in seed treatments indicated a microbial community shaped by plant‐derived exudates rather than fruit decomposition. Both taxa are associated with plant growth promotion and nitrogen cycling (Compant *et al*., 2019; Yadav *et al*., 2021).

Although functional changes are only predicted based on amplicon data, the soil microbial functional potential reflects shifts in soil microbial community composition, reinforcing that fruit decomposition alters microbial structure and metabolic potential. The fruit treatment microbiome was enriched in decomposition‐related pathways, whereas the seed treatment was associated with nitrogen cycling. We suggest a strong microbial nitrogen regulation (Kuypers *et al*., 2018), which could be beneficial for plant growth. Functional enrichments in mammalian and human gut metabolism under fruit treatment likely reflect substrate‐ and microsite‐driven selection for facultative anaerobes and fermentative lineages that occur in both guts and plant and soil habitats. Abundant simple carbohydrates from decomposing tomato fruits could create oxygen‐limited microzones, which favor enteric‐like metabolisms. However, since FAPROTAX is taxonomy‐based, these labels indicate the ecological affinity of the taxa detected, not direct functional measurements.

### The effect of fruit decomposition on the seedling germination and growth

Our findings highlight that fruit decomposition affects plant performance, notably by reducing germination rates. Fruit decomposition may introduce microbial competition and nutrient imbalances that affect seed germination. Furthermore, fruit pulp contains allelopathic compounds such as alkaloids and phenolics, which can inhibit enzymes and water uptake essential for seed dormancy release (Baskin & Baskin, 2000; Al‐Khayri *et al*., 2023). However, it is possible that the observed reduction in germination is only temporary. The allelopathic compounds may delay germination rather than completely prevent it, and over time, these substances could degrade, allowing seeds to eventually germinate. We could not observe this potential long‐term effect, as the 18‐d observation period was too short. Despite reduced germination, tomato seedlings had significantly longer shoots in the fruit treatments, whereas chili seedlings had significantly shorter shoots. Nutrient release from decomposing fruit may promote shoot elongation in tomatoes, whereas in chili, microbial interactions or inhibitory compounds from fruit decomposition may suppress growth. This suggests that fruit decomposition may create localized conditions that stimulate or inhibit seedling growth (Kato‐Noguchi & Tanaka, 2003; Arin & Arabaci, 2019). Root length remained consistent in chili and tomato, indicating that root‐microbe interactions and intrinsic plant developmental constraints influence root growth more, whereas shoot elongation can be influenced by external conditions.

### The effect of fruit decomposition on phyllosphere and rhizosphere microbiomes and predicted functions

We demonstrated – particularly in the chili experiment – that a considerable fraction of the phyllosphere and a smaller amount of the rhizosphere can be assigned to the fruit source, consistent with microbial coalescence from soil, seed and fruit to plant‐associated habitats seedlings (Rillig *et al*., 2016; Rochefort *et al*., 2021). Soil sources, especially at later time points, remain the dominant contributors to the phyllosphere and rhizosphere microbiome and have a similar proportion between the fruit and seed treatments. The seed treatment in chili shows a very similar pattern between the phyllosphere and rhizosphere. By contrast, tomato exhibits a smaller fruit‐assigned fraction, which we correlate to sequencing depth and platform differences between experiments. Overall, these results support the idea of microbial coalescence and provide additional context for understanding the origin of microbial communities associated with the emerging plant.

Phyllosphere microbial diversity in both tomatoes and chili was significantly lower in fruit treatments than in seed treatments, whereas rhizosphere diversity remained stable. The phyllosphere microbiome is likely more influenced by fruit‐derived microorganisms, which may outcompete or suppress others, whereas rhizosphere communities are more stable because of plant–root–microbe interactions that buffer external shifts (Shade *et al*., 2017). Community composition in both the phyllosphere and rhizosphere differed significantly among treatments, indicating that diversity metrics alone may overlook key shifts. In the tomato phyllosphere, fruit treatment enriched *Methylophilus* and *Ralstonia*, associated with nitrogen cycling and altered nutrient dynamics (Bai *et al*., 2015), whereas seed treatments favored *Aeromicrobium* and *Chryseobacterium*, taxa linked to plant‐beneficial functions (Hardoim, 2019; Rochefort *et al*., 2021; Zeng *et al*., 2023). In the tomato rhizosphere, fruit treatment enriched *Flavobacterium*, known for organic matter degradation (Kolton *et al*., 2013; Máté *et al*., 2022), while seed treatments enriched *Devosia*, a nitrogen‐fixing genus (Madhaiyan *et al*., 2015; Negi *et al*., 2022). In the chili phyllosphere, fruit treatment enriched *Rhizobium*, suggesting enhanced nitrogen fixation and potential plant growth benefits (Zgadzaj *et al*., 2016). The strong presence of *Sandaracinaceae* in the tomato and chili seed treatments suggests a conserved selection process for this taxon. Furthermore, the increase in *Stenotrophomonas* in the chili rhizosphere points to potential biocontrol capabilities due to its antagonistic activity against pathogens (Ryan *et al*., 2009; Sharma *et al*., 2024). Overall, taxa enriched in the fruit treatment are likely to be more involved in organic matter decomposition, whereas seed treatment enriches microbes that interact more closely with plant metabolism.

Functional capacities differed between fruit and seed treatments in both chili and tomato, aligning with taxonomic shifts and potentially influencing plant fitness and nutrient cycling (Louca *et al*., 2018). The phyllosphere in fruit treatment shows enrichment in pathogen metabolism and degradation pathways, suggesting a more decomposer‐driven microbiome, active degradation and use of plant exudates (Chistoserdova, 2011; Krishnamoorthy *et al*., 2021). Fruit treatment enriched sulfur and nitrogen cycling in the rhizosphere, which may affect plant–microbe interactions related to nutrient uptake. Moreover, seed treatment promotes methanotrophy and nitrogen fixation, which are essential for plant growth (Malik *et al*., 1997; Iguchi *et al*., 2015).

### Conclusion

This study highlights the effects of fruit decomposition on soil abiotic and biotic composition, plant performance, and plant microbiomes including their functions. Fruit decomposition influences soil nutrient dynamics, mostly in increased potassium availability and pH level. The low germination rate in the fruit treatment suggests growth inhibitory substances that may inhibit the growth of seedlings; however, the growth response of shoots and roots was crop‐specific. Microbial community responses to fruit decomposition were evident in both plants. In chili and tomato, phyllosphere microbiomes showed lower bacterial diversity due to fruit decomposition, and were taxonomically and functionally different from the seed treatment. Fruit decomposition did not affect rhizosphere microbial diversity, but community composition and functional profiles changed drastically. This indicates that early colonization of fruits or seeds by microbes plays a crucial role in shaping plant‐associated microbiomes and soil microbial networks. In conclusion, this study underscores the profound impact of fruit decomposition on soil health, plant performance, and microbiomes. We emphasize the complex interactions between fruit decomposition, microbial communities, and soil nutrient dynamics, demonstrating how decomposition alters microbial structure and functional potential. While our experimental design provides a direct test of the effects of fruit decomposition on the tomato and chili plant microbiome and fitness, future research should focus on experimental validation based on our findings, for example disentangling chemistry vs microbe effects and following microbial transmission from fruit to seedling. Additionally, FAPROTAX calls should be confirmed with targeted assays for example metabolites, enzyme activities, metagenomes, and gene expression to quantify both the direction and effect size of functional changes.

## Competing interests

None declared.

## Author contributions

AA conceptualized and designed the experiments. DH, MAH, and WK performed experiments and laboratory work. AA, DKR, and DH performed bioinformatics analyses. LS and AM provided the facilities for sequencing the samples of the tomato study. AA, DKR, DH, and DK wrote the first draft of this manuscript. AJMT, LS, and AM contributed to the interpretation of results. All authors have read and approved the final manuscript. DH and DKR contributed equally to this work.

## Disclaimer

The New Phytologist Foundation remains neutral with regard to jurisdictional claims in maps and in any institutional affiliations.

## Supporting information


**Notes S1** Analysis of soil physicochemical properties.
**Notes S2** DNA extraction, amplicon library preparation, and sequencing.
**Table S1** Fold change analysis of soil chemical properties and pH between seed and fruit treatments.
**Table S2** Statistical summary of microbial diversity and soil community composition in tomato and chili under fruit and seed treatments.
**Table S3** Statistical summary of microbial diversity and community composition in phyllosphere and rhizosphere microbiomes of tomato and chili.
**Table S4** PERMANOVA summary of functional composition in phyllosphere and rhizosphere microbiomes of tomato and chili.
**Table S5** Differentially enriched predicted bacterial functions across compartments and treatments in tomato and chili.
**Table S6** Mean source contributions inferred by SourceTracker2 for tomato and chili phyllosphere and rhizosphere microbiomes under fruit and seed treatments.
Please note: Wiley is not responsible for the content or functionality of any Supporting Information supplied by the authors. Any queries (other than missing material) should be directed to the *New Phytologist* Central Office.

## Data Availability

The datasets generated and/or analyzed during the current study are available in the ‘SRA NCBI’ repository under the Bioproject ID PRJNA1254098. The analysis code used in this study is available on Zenodo and can be accessed from the following link: 10.5281/zenodo.17337997.
