# Supplementary material for: Fruit function beyond dispersal: effect of fruit decomposition on the plant microbiome assembly
Source: New Phytol. 2025 Nov 17;249(3):1442–55. doi: 10.1111/nph.70698 (PMC12780309; doi:10.1111/nph.70698)
Supplement: Supplementary file 1 — Notes S1 Analysis of soil physicochemical properties. Notes S2 DNA extraction, amplicon library preparation, and sequencing. Table S1 Fold change analysis of soil chemical properties and pH between seed and fruit treatments. Table S2 Statistical summary of microbial diversity and soil community composition in tomato and chili under fruit and seed treatments. Table S3 Statistical summary of microbial diversity and community composition in phyllosphere and rhizosphere microbiomes of tomato and chili. Table S4 PERMANOVA summary of functional composition in phyllosphere and rhizosphere microbiomes of tomato and chili. Table S5 Differentially enriched predicted bacterial functions across compartments and treatments in tomato and chili. Table S6 Mean source contributions inferred by SourceTracker2 for tomato and chili phyllosphere and rhizosphere microbiomes under fruit and seed treatments. Please note: Wiley is not responsible for the content or functionality of any Supporting Information supplied by the authors. Any queries (other than missing material) should be directed to the New Phytologist Central Office. [file NPH-249-1442-s001.pdf]

***New Phytologist* Supporting Information**

Article title:
Fruit function beyond dispersal: effect of fruit decomposition on plant microbiome assembly

Authors:
Daniel Hoefle, Dinesh Kumar Ramakrishnan, Marie-Antoinette Holländer, Denis Kiplimo, William Konzag , Leonardo Schena, Antonino Malacrinò, Ayco J. M. Tack, Ahmed Abdelfattah

Article acceptance date:
09 October 2025

The following Supporting Information is available for this article:

Notes S1: Analysis of Soil physicochemical properties

Notes S2: DNA extraction, amplicon library preparation and sequencing

Table S1: Fold change analysis of soil chemical properties and pH between seed and fruit treatments.

Table S2: Statistical summary of microbial diversity and soil community composition in tomato and chili under fruit and seed treatments.

Table S3: Differentially enriched predicted bacterial functions across compartments and treatments in tomato and chili.

Table S4: Statistical summary of microbial diversity and community composition in phyllosphere and rhizosphere microbiomes of tomato and chili.

Table S5: Statistical summary of functional composition in phyllosphere and rhizosphere microbiomes of tomato and chili.

Table S6: Mean source contributions inferred by SourceTracker2 for tomato and chili phyllosphere and rhizosphere microbiomes under fruit and seed treatments.

Notes S1

Soil physicochemical properties analysis

1.1. Organic carbon

The organic carbon content in the soil was determined by measuring both the total inorganic carbon content (TIC) and the total carbon content (TC). To determine TC, soil samples were burned at 1200 °C and measured with an element analyzer (multi EA 4000, Analytik Jena, Germany). For TIC measurement, phosphoric acid was added to the soil samples, followed by the measurement at 80 °C in the same element analyzer. The organic carbon content was then calculated by subtracting the TIC from the TC. Measurement of total kjeldahl nitrogen (TKN), excluding nitrates and nitrites, was conducted by Kjeldahl method.

1.2. Plant available Mg, S, P and K

Measurements of plant available magnesium, sulfur, phosphorus and potassium are based on VDLUFA methods book volume 1. The extraction of plant available sulfur and magnesium was carried out by adding CaCl2 solution (0.0125 M) to fresh and homogenized soil samples (excluding chili pepper fruit and seeds). The content of plant available sulfur and magnesium in the soil was determined using an atomic absorption spectrometer (AAS; ContrAA700, Autosampler AS-FD, Analytik Jena, Germany). For pH-value detection, the same extract was used. Plant available phosphorus and potassium were determined from sieved dry soil. The extraction of these nutrients was performed by using the double lactate method. Plant available phosphorus was subsequently detected using the molybdenum blue method in conjunction with an autoanalyzer (FIA compact, MLE, Germany), while plant available potassium was detected by an atomic absorption spectrometer (AAS; ContrAA700, Autosampler AS-FD, Analytik Jena, Germany).

1.3. Concentrations of Mg, Ca, Cu, Fe, Mn, Na, P, S and Zn

Measurements of total concentrations of magnesium (Mg), calcium (Ca), copper (Cu), iron (Fe), manganese (Mn), sodium (Na), phosphorus (P), sulfur (S), and zinc (Zn) in the soil were conducted

from dried and ground soil samples. The extraction and subsequent measurement were carried out based on the VDLUFA methods book volume 3. The extraction was done with nitric acid in an UltraClave IV microwave (Milestone). An inductively coupled plasma optical emmission spectroscope (ICP-OES; iCAP6300 Duo, Thermo Scientific) was subsequently used to determine the nutrient contents.

Notes S2

DNA extraction, amplicon library preparation and sequencing

2.1. Tomato

To determine the bacterial composition, seed (3 seeds), fruit (0.2 g), shoot (total) and root (total) samples were separately placed in sterile plastic bags containing 1 mL of 0.9 % NaCl and subsequently homogenized using a mortar and pestle. The liquid phase of the homogenate was transferred into a lysis tube and DNA extraction performed using the FastPrep™ Spin Kit for Soil (MP Biomedicals, CA, USA) following the manufacturer’s instructions. The DNA was kept at -20 ℃ until further processing. To generate an amplicon library for Illumina sequencing we amplified the 16S rRNA V4 gene region using the universal bacterial primer pair 515f and 806r including Illumina adapters (www.illumina.com). Each PCR reaction had a total volume of 15 µL, including 2 µL DNA for plant samples and 1 µL DNA for soil, and 7.5 µL 2x KAPA HiFi HotStart Mix (Roche, Basel, CH), 0.4 µL of each primer [10 µM], 0.75 µL mPNA and pPNA [5 µM] (PNA Bio Inc, CA, USA), and 3.2/4.2 µL PCR grade water (depending on the sample). The peptide nucleic acids (PNAs) PCR clamps were used to block the amplification of mitochondrial and plastid 16S rRNA during PCR amplification. PCR reactions were performed at the following conditions: initial denaturation at 98 ℃ for 3 minutes, and for 30 cycle 95 ℃ for 30 seconds, 78 ° C for 5 seconds, 58 ℃ for 30

seconds, and 72 ° C for 30 seconds. At last, a final extension step at 72 ℃ for 3 minutes. The PCR product was purified using AMPure XP magnetic beads (Beckman Coulter, CA, USA) following the manufacturers protocol. To add the Illumina sequencing adapters we performed a second PCR in a total of 25 µL reaction mix. Each reaction contained 5 µL of the purified PCR product of the first PCR as DNA template, 12.5 µL 2x KAPA HiFi HotStart Mix (Roche, Basel, CH), 2.5 µL of each Nextera XT index primer pair (N7XX and S5XX, Illumina, CA, USA) and 5 µL PCR grade water. The thermocycler' s program was 3 minutes at 95 ℃, and 8 cycles of at 95 ℃ for 30 seconds, 55 ℃ for 30 seconds, 72 ° C for 30 seconds ending with a final extension step at 72 ℃ for 5 minutes. Successful amplification was confirmed by gel electrophoresis using a 1 % gel with 1X TAE buffer. All samples were purified using AMPure Magnetic Beads (Beckman Coulter, USA) and subsequently DNA concentration was quantified using the Qubit dsDNA HS Assay Kit on a Qubit 4 fluorimeter (Thermo Fisher Scientific, Waltham, USA). The DNA of all samples were normalized and pooled and sequenced on an Illumina MiSeq (2 x 300 bp) following the manufacturer's instructions.

2.2. Chili

To analyse the bacterial community of different samples from chili plants, 0.4 g of each sample tissue (seed, fruit, soil, shoot and root) was sampled. Prior to DNA extraction, seed, fruit, shoot and root samples were ground in liquid nitrogen by using a mortar and pestle. DNA extraction was performed using the FastDNA™ SPIN Kit for Soil (MP Biomedicals, CA, USA). The DNA was kept at -20 ℃ until further processing. For amplicon library preparation the DNA was diluted with nuclease-free PCR-grade water. DNA from soil samples was diluted 1:10 or 1:100. DNA from root and shoot samples was diluted 1:10, and extracted DNA from seeds and fruits was used

undiluted. For amplification of the bacterial 16S rDNA V4 a PCR reaction mix consisting of 8.35 µL PCR-grade water, 1 µL DNA template, 12.5 µL 2x repliQa HiFi ToughMix (Quanta Biosciences, MA, USA), each 0.375 µL of mPNA [50 µM] and pPNA [50 µM], and each 1.2 µL of 515f [5 µM] and 806r primers [5µM] with attached barcodes (designed by the EarthMicrobiomeProject, www.earthmicrobiome.org) was prepared. For every sample, a unique combination of forward and reverse barcode primers was used. The DNA was amplified by using a thermocycler (TProfessional BASIC, Biometra, Germany) with the following settings: pre-heating at 98 °C, initial denaturation at 98 °C for 10 seconds, followed by 35 cycles at 98 °C for 10 seconds, 78 °C for 5 seconds, 53 °C for 15 seconds, and extension at 68 °C for 2 seconds. Successful amplification was verified by gel electrophoresis using a 1 % agarose gel with 1x TAE buffer. The PCR products were purified using SPRIselect magnetic beads (Beckman Coulter, CA, USA) following the manufacturers protocol. The DNA concentration of each sample was measured with a Qubit 4 fluorimeter (Thermo Fisher Scientific, MA, USA). An amplicon pool was prepared by mixing all PCR products with an equal molarity and the pool was sent for sequencing on an Illumina NovaSeq 6000 (2 x 250) to Novogene, Munich, Germany.

Table S1 Summary of fold change (FC) analysis for soil chemical properties and pH between seed and fruit treatments in chili (Capsicum annuum L.) cultivation. FC_GMR = geometric mean ratio of seed vs. fruit; Log_2FC = log_2-transformed FC_GMR; CI_low and CI_high = lower and upper bounds of the 95% confidence interval; p-value = unadjusted significance; FC_mean and FC_median = arithmetic mean and median fold changes; p_adj = Benjamini–Hochberg adjusted p-value. For pH, ΔpH = difference between seed and fruit mean pH, H^+ FC = fold change in proton concentration (seed vs. fruit), and Seed_mean_pH and Fruit_mean_pH = mean pH values per treatment.

	FC_GMR	Log$_2$FC	CI_low	CI_high	p_value	FC_mean	FC_median	p_adj
**C org.**	1.1073	0.1470	0.9507	1.2896	0.1391	1.1107	1.1012	0.1391
**Magnesium**	1.0638	0.0892	0.9927	1.1399	0.0685	1.0631	1.0666	0.0913
**Potassium**	0.8670	-0.2060	0.8111	0.9267	0.0029	0.8665	0.8727	0.0115
**Sulfur**	1.1224	0.1665	1.0301	1.2229	0.0207	1.1235	1.1153	0.0413
	**Delta_pH**	**Hplus_FC_Seed_vs_Fruit**			**Seed_mean_pH**		**Fruit_mean_pH**	
**pH**	-0.0475	1.12			6.42		6.47	

Table S2 Statistical summary of microbial diversity and community composition in the soil microbiomes of tomato (Solanum lycopersicum L.) and chili (Capsicum annuum L.) under fruit and seed treatments. P-values for species richness and Shannon diversity were obtained from ANOVA testing the effect of treatment, time, and their interaction. Community and functional composition were assessed using PERMANOVA based on Bray–Curtis dissimilarities to test the interactive effect of treatment and time; P-values were fdr-adjusted. Partial R^2 values indicate the proportion of variation explained by treatment effects. Significant values ($P < 0.05$) are shown in bold.

ANOVA	Microbial Diversity	Tomato		Chilli	
		Richness (P-value)	Shannon (P-value)	Richness (P-value)	Shannon (P-value)
Treatment	Soil	**0.012**	**0.012**	0.854	0.593
Time	Soil	**0.008**	**0.003**	0.119	0.053
Treatment * Time	Soil	0.258	0.349	0.409	0.661
PERMANOVA	Community composition	Tomato		Chilli	
		P-value	R^2	P-value	R^2
Treatment * Time	Soil	**0.001**	0.093	**0.004**	0.050
PERMANOVA	Functional composition	Tomato		Chilli	
		P-value	R^2	P-value	R^2
Treatment * Time	Soil	**0.002**	0. 105	0.401	0.033

Table S3 Differentially enriched predicted bacterial functions in tomato (Solanum lycopersicum L.) and chili (Capsicum annuum L.) seedlings across soil, phyllosphere, and rhizosphere compartments under fruit and seed treatments. Functional annotations were inferred from CSS-

normalized 16S rRNA gene data using FAPROTAX and tested for differential abundance with DESeq2. Columns report log$_2$-transformed fold change (log$_2$FoldChange), adjusted P-value (padj), and the treatment with higher relative abundance (Regulation). Analyses were performed separately for each host species and compartment; only functions with padj < 0.05 and meeting compartment-specific log$_2$ fold-change (LFC) thresholds are shown (tomato: soil T2 $|LFC| \geqslant 1.0$, phyllosphere $\geqslant 0.5$, rhizosphere $\geqslant 2.0$; chili: soil T2 $\geqslant 0.5$, phyllosphere/rhizosphere $\geqslant 1.0$). Significant results ($P < 0.05$) are shown in bold.

**Tomato soil**	**log2FoldChange**	**padj**	**Regulation**
aerobic ammonia oxidation	2.709039	1.54E-09	Fruit
human gut	2.974226	2.20E-07	Fruit
human pathogens pneumonia	2.671389	1.17E-20	Fruit
intracellular parasites	2.398262	6.63E-13	Fruit
mammal gut	2.974226	2.20E-07	Fruit
methanol oxidation	2.273856	6.77E-17	Fruit
methylotrophy	1.772236	1.96E-14	Fruit
nitrification	2.662367	1.29E-10	Fruit
nitrogen fixation	1.053085	1.94E-04	Fruit
nonphotosynthetic cyanobacteria	1.461469	2.16E-06	Fruit
predatory or exoparasitic	1.717757	2.63E-10	Fruit
aromatic compound degradation	-1.061248	2.94E-05	Seed
denitrification	-2.826043	4.56E-28	Seed
nitrate denitrification	-2.826043	4.56E-28	Seed
nitrate reduction	-1.291452	2.81E-11	Seed
nitrate respiration	-1.980205	2.86E-21	Seed
nitrite denitrification	-2.826043	4.56E-28	Seed
nitrite respiration	-2.589512	6.42E-28	Seed
nitrogen respiration	-1.980205	2.86E-21	Seed
nitrous oxide denitrification	-3.036238	4.06E-34	Seed
plant pathogen	-3.036637	4.06E-34	Seed
**Tomato phyllosphere**	**log2FoldChange**	**padj**	**Regulation**
human pathogens pneumonia	1.841229	0.00133201	Fruit
methanol oxidation	1.888903	0.00117939	Fruit
methylotrophy	1.51409	0.00415793	Fruit
ureolysis	1.183111	0.02222588	Fruit
aromatic compound degradation	-0.809817	0.01707914	Seed
**Tomato rhizosphere**	**log2FoldChange**	**padj**	**Regulation**
dark thiosulfate oxidation	5.281501	2.82E-04	Fruit
intracellular parasites	2.637756	4.05E-09	Fruit

xylanolysis	2.153714	8.56E-05	Fruit
denitrification	-3.206396	8.88E-10	Seed
nitrate denitrification	-3.206396	8.88E-10	Seed
nitrite denitrification	-3.206396	8.88E-10	Seed
nitrite respiration	-2.489618	4.46E-06	Seed
nitrous oxide denitrification	-3.764443	1.50E-06	Seed
plant pathogen	-2.511486	2.38E-04	Seed
**Chili soil**	**log2FoldChange**	**padj**	**Regulation**
aliphatic non methane hydrocarbon degradation	0.6385325	0.027707911	Fruit
aromatic hydrocarbon degradation	0.6662372	0.026261072	Fruit
hydrogenotrophic methanogenesis	1.7478272	0.027707911	Fruit
methanogenesis	1.7478272	0.027707911	Fruit
methanogenesis by CO2 reduction with H2	1.7721967	0.027707911	Fruit
human gut	-2.327883	0.000184982	Seed
mammal gut	-2.327883	0.000184982	Seed
nitrate reduction	-0.812058	0.000476842	Seed
**Chili phyllosphere**	**log2FoldChange**	**padj**	**Regulation**
aliphatic non methane hydrocarbon degradation	1.144098	2.88E-03	Fruit
aromatic compound degradation	1.247497	4.90E-05	Fruit
aromatic hydrocarbon degradation	1.234814	1.50E-03	Fruit
ligninolysis	3.229676	6.56E-03	Fruit
plant pathogen	1.049628	1.84E-03	Fruit
cellulolysis	-1.581071	2.99E-03	Seed
methanotrophy	-1.145535	9.75E-19	Seed
nonphotosynthetic cyanobacteria	-1.079197	1.84E-03	Seed
photoheterotrophy	-1.939186	1.83E-02	Seed
phototrophy	-1.952117	1.82E-02	Seed
**Chili rhizosphere**	**log2FoldChange**	**padj**	**Regulation**
chitinolysis	1.388569	1.92E-05	Fruit
dark oxidation of sulfur compounds	1.292959	1.65E-03	Fruit
denitrification	1.056644	1.38E-06	Fruit
ligninolysis	2.520282	1.45E-02	Fruit
nitrate denitrification	1.056644	1.38E-06	Fruit
nitrate respiration	1.274175	3.15E-24	Fruit
nitrite denitrification	1.056644	1.38E-06	Fruit
nitrogen respiration	1.274175	3.15E-24	Fruit
plant pathogen	1.991329	4.48E-05	Fruit
hydrogenotrophic methanogenesis	-2.041131	7.23E-03	Seed
methanogenesis	-2.041131	7.23E-03	Seed
methanogenesis by CO2 reduction with H2	-2.041131	7.23E-03	Seed

Table S4 Statistical summary of microbial diversity and community composition in the phyllosphere and rhizosphere microbiomes of tomato (Solanum lycopersicum L.) and chili (Capsicum annuum L.) under fruit and seed treatments. P-values for species richness and Shannon diversity were obtained from ANOVA. Community composition was evaluated using PERMANOVA based on Bray–Curtis dissimilarities to test treatment effects. P-values were fdr-adjusted. Partial R^2 values indicate the proportion of variation explained by treatment effects. Significant results ($P < 0.05$) are shown in bold.

PERMANOVA	Microbial Diversity	Tomato		Chilli	
		Richness (P-value)	Shannon (P-value)	Richness (P-value)	Shannon (P-value)
Treatment	Phyllosphere	0.127	**0.036**	**<0.001**	**<0.001**
Treatment	Rhizosphere	0.311	0.315	0.168	0.278
PERMANOVA	Community composition	Tomato		Chilli	
		P-value	R^2	P-value	R^2
Treatment	Phyllosphere	**0.001**	0.166	**0.001**	0.453
Treatment	Rhizosphere	**0.001**	0.195	**0.008**	0.175

Table S5 Statistical summary of functional composition in the phyllosphere and rhizosphere microbiomes of tomato (Solanum lycopersicum L.) and chili (Capsicum annuum L.) under fruit and seed treatments. Functional community composition was evaluated using PERMANOVA based on Bray–Curtis dissimilarities to test treatment effects. P-values were fdr-adjusted. Partial R^2 values indicate the proportion of variation explained by treatment effects. Significant results ($P < 0.05$) are shown in bold.

PERMANOVA	Functional composition	Tomato		Chilli	
		P-value	R^2	P-value	R^2
Treatment	Phyllosphere	**0.020**	0.133	**0.001**	0.458
Treatment	Rhizosphere	**0.001**	0.374	**0.001**	0.200

Table S6 Mean microbial source contributions inferred by SourceTracker2 for tomato (Solanum lycopersicum L.) and chili (Capsicum annuum L.) seedling microbiomes under fruit and seed treatments. Mean proportions (mean_prop) represent the inferred contributions of fruit, seed, and soil sources to the phyllosphere and rhizosphere microbiomes, estimated on 16S rRNA gene amplicon data. Columns show contributions from fruit or seed, baseline soil (Soil_T0), and soils sampled at later times (Soil_T1, Soil_T2), as well as "Unknown," which represents reads not attributable to modeled sources. Values indicate mean proportions across biological replicates; higher values correspond to greater estimated source contributions to the sink community. Sampling times T0-T2 denote the temporal progression of soil sampling.

**Tomato fruit treatment**	Phyllosphere					Rhizosphere				
Source	Fruit	Soil_T0	Soil_T1	Soil_T2	Unknown	Fruit	Soil_T0	Soil_T1	Soil_T2	Unknown
mean_prop	0.0523	0.0286	0.0776	0.0676	0.774	0.00662	0.0881	0.169	0.18	0.557
**Tomato seed treatment**	Phyllosphere					Rhizosphere				
Source	Seed	Soil_T0	Soil_T1	Soil_T2	Unknown	Seed	Soil_T0	Soil_T1	Soil_T2	Unknown
mean_prop	0.0115	0.103	0.102	0.21	0.574	0.00178	0.181	0.134	0.388	0.295
**Chili fruit treatment**	Phyllosphere					Rhizosphere				
Source	Fruit	Soil_T0	Soil_T1	Soil_T2	Unknown	Fruit	Soil_T0	Soil_T1	Soil_T2	Unknown
mean_prop	0.246	0.114	0.224	0.111	0.306	0.127	0.144	0.213	0.17	0.346
**Chili seed treatment**	Phyllosphere					Rhizosphere				
Source	Seed	Soil_T0	Soil_T1	Soil_T2	Unknown	Seed	Soil_T0	Soil_T1	Soil_T2	Unknown
mean_prop	0.0324	0.221	0.221	0.193	0.333	0.0278	0.235	0.226	0.217	0.294
